# Traces of Paleolithic expansion in the Nivkh gene pool based on data on autosomal SNP and Y chromosome polymorphism

**DOI:** 10.18699/vjgb-24-73

**Published:** 2024-10

**Authors:** V.N. Kharkov, N.A. Kolesnikov, L.V. Valikhova, A.A. Zarubin, A.L. Sukhomyasova, I.Yu. Khitrinskaya, V.A. Stepanov

**Affiliations:** Research Institute of Medical Genetics, Tomsk National Research Medical Center of the Russian Academy of Sciences, Tomsk, Russia; Research Institute of Medical Genetics, Tomsk National Research Medical Center of the Russian Academy of Sciences, Tomsk, Russia; Research Institute of Medical Genetics, Tomsk National Research Medical Center of the Russian Academy of Sciences, Tomsk, Russia; Research Institute of Medical Genetics, Tomsk National Research Medical Center of the Russian Academy of Sciences, Tomsk, Russia; M.K. Ammosov North-Eastern Federal University, Yakutsk, Russia; Research Institute of Medical Genetics, Tomsk National Research Medical Center of the Russian Academy of Sciences, Tomsk, Russia; Research Institute of Medical Genetics, Tomsk National Research Medical Center of the Russian Academy of Sciences, Tomsk, Russia

**Keywords:** gene pool, human populations, genetic diversity, genetic components, Y chromosome, Nivkhs, генофонд, популяции человека, генетическое разнообразие, генетические компоненты, Y-хромосома, нивхи

## Abstract

The Nivkhs are a small ethnic group indigenous of the Russian Far East, living in the Khabarovsk Territory and on Sakhalin Island, descending from the ancient inhabitants of these territories. In the Nivkhs, a specific Sakhalin-Amur anthropological type is prevalent. They are quite isolated, due to long isolation from contacts with other peoples. The gene pool of the Nivkhs and other Far Eastern and Siberian populations was characterized using a genome-wide panel of autosomal single-nucleotide polymorphic markers and Y chromosome haplogroups. Bioinformatic processing of frequencies of autosomal SNPs, Y chromosome haplogroups and YSTR haplotypes showed that the Nivkh gene pool is very different from the other populations’. Analysis of the SNP frequencies using the PCA method divided the Far Eastern populations in full accordance with the territories of their residence into the northern group of the Chukchi and Koryaks and the southern group, including the Nivkhs and Udege. The remoteness of the Nivkhs coincides with their geographic localization, with the Nivkhs and Udege demonstrating the greatest kinship. The Nivkhs have a specific component of their gene pool, which is present with much less frequency in the Udege and Transbaikal Evenks. According to the IBD blocks, the genotypes of the Nivkhs show a very small percentage of coincidence with the Udege, Koryaks, Evenks and Chukchi, the value of which is the lowest compared to the IBD blocks among all other Siberian populations. The Nivkh-specific composition of haplogroups and YSTR haplotypes was shown. In the Nivkhs, the C2a1 haplogroup is divided into three sublines, which have a fairly ancient origin and are associated with the ancestors of modern northern Mongoloids. The Nivkh haplogroup O2a1b1a2a-F238 is found among residents of China and Myanmar. The Q1a1a1-M120 line is represented among the Nivkhs, Koryaks, Evenks and Yukaghirs. Phylogenetic analysis of individual Y chromosomal haplogroups demonstrated the closeness of the Nivkh gene pool with the ancient population of the Amur and Okhotsk regions, the Koryaks, the Tungus peoples and the population of Southeast Asia. The Nivkh gene pool confirms the relative smallness of their ancestral groups without mixing with other populations.

## Abstract (ru)

Нивхи – малочисленный коренной народ Дальнего Востока, проживающий на территории Хабаровского края и острова Сахалин, который относится к потомкам древнего населения этих территорий. У нивхов преобладает специфичный сахалино-амурский антропологический тип. Они являются достаточно обособленными за счет длительной изоляции от контактов с другими народами. Генофонд нивхов охарактеризован по полногеномной панели аутосомных однонуклеотидных полиморфных маркеров и гаплогруппам Y-хромосомы в сравнении с другими дальневосточными и сибирскими популяциями. Биоинформатическая обработка частот аутосомных SNP, гаплогрупп Y-хромосомы и YSTR-гаплотипов показала, что генофонд нивхов существенно отличается от генофондов других популяций. При анализе частот SNP методом PCA дальневосточные популяции располагаются в полном соответствии с территориями их проживания и делятся на северную группу чукчей и коряков и южную, включающую нивхов и удэгейцев. Удаленность нивхов совпадает с их географической локализацией, при этом нивхи и удэгейцы демонстрируют наибольшее родство. У нивхов выделяется специфичный для них компонент генофонда, который с гораздо меньшей частотой присутствует у удэгейцев и забайкальских эвенков и бурятов-А. По IBD-блокам генотипы нивхов демонстрируют очень небольшую долю совпадения с удэгейцами, коряками, эвенками и чукчами, значение которых является самым низким по сравнению с IBD-блокам между другими сибирскими популяциями. Показан специфичный для нивхов состав гаплогрупп и YSTR-гаплотипов. Гаплогруппа C2a1 у нивхов разделена на три сублинии, которые имеют достаточно древнее происхождение и связаны с предками современных северных монголоидов. Нивхская гаплогруппа O2a1b1a2a-F238 есть у жителей Китая и Мьянмы. Линия Q1a1a1-M120 в исследованных в данной работе выборках представлена у нивхов, коряков, эвенков и юкагиров. Филогенетический анализ отдельных Y-хромосомных гаплогрупп демонстрирует близость генофонда нивхов с коряками и тунгусскими народами, а также родство в меньшей степени с древним населением Приамурья и Приохотья и населением Юго-Восточной Азии. Генофонд нивхов подтверждает относительную малочисленность их предковой группы без смешения с другими популяциями.

## Introduction

The Nivkh people are a small ethnic group that lives in the Far Eastern regions of Sakhalin Island and the lower Amur Basin. In 2022, there were about 3,842 Nivkh people. They self-identify as nivkhgu. Neighboring ethnic groups call them Gilyak or Gilyami, and the Russians adopted this name, calling them Gilak. In Tungusic and Manchu languages, the word “Gilyaki” means “people who move with the help of oar-powered boats”.

Based on their territory, the Nivkhs can be divided into two groups: the island group (Sakhalin) and the mainland group. In the past, they occupied a much larger territory. On the mainland, their settlement area extended from the Amur River to the Uda Basin, and on Sakhalin, they lived along the western and eastern coastlines and at the mouth of the Poronai River. Nowadays, the Sakhalin Nivkhs live in the northern part of the island and the Tym River basin. In the mainland, the Nivkh people are concentrated in two districts of the Khabarovsk Territory: Nikolaevsky and Ulchsky. They speak a language called Nivkh, which has two dialects: Amur and East Sakhalin. Nivkh is an isolated language, along with Ket and Yukaghir. It was previously classified as part of the Paleoasiatic language family due to its unclear genealogical origins. A strong relationship between Nivkh and the Chukchi-Kamchatkan languages was found in the work of M.D. Fortescue (2011).

The Nivkh people are direct descendants of the ancient population that inhabited Sakhalin and the lower reaches of the Amur River in the past. They are part of the Paleo-Asiatic group of the Mongoloid race, and their anthropological type is similar to that of the Sakhalin-Amur people, which can also be found among the Ulchi people. Together with the Chukchi, Koryaks, and other people of Northeastern Siberia, the Nivkhs belong to the Paleoasian group. There is a theory that the ancestors of the modern Nivkhs, as well as the Eskimo and Native American peoples, were all links in the same ethnic chain that once covered the northwestern coast of the Pacific Ocean. The modern appearance of the Nivkhs has been significantly influenced by their cultural and ethnic interactions with the Tungus-Manchu, Ainu, and Japanese people (The Peoples of Russia, 1994; Sulyandziga et al., 2003; Peoples of North-East Siberia, 2010).

The data obtained from genotyping high-density microarrays for autosomal SNPs in the Nivkhs and other Far Eastern and Siberian indigenous peoples allow us to more accurately describe their gene pool composition, identify common haplotype blocks, and homozygosity patterns compared to limited sets of DNA markers. Genotyping a larger set of specific Y- chromosome SNPs enables a more detailed characterization of the molecular phylogenetic structure of Y-haplogroups. Modern bioinformatics methods for individual genotype analysis allow us to characterize the gene pool of the studied samples as thoroughly as possible using various techniques.

There are a vast number of Single Nucleotide Polymorphisms (SNPs) in the human genome, which makes them an effective tool for analyzing genetic relationships between populations. Modern population genetics has various marker systems, including autosomal and homologous DNA markers that determine the phylogeny of the Y-chromosome and mitochondrial DNA haplogroups.

A specific feature of the mitochondrial gene pools in all Primorye populations is the presence of mtDNA lines belonging to haplogroup Y. The maximum frequencies of these lines were noted in the Sakhalin Nivkh (66.1 %) and Ulchi populations (37.9 %). The frequency of this line is also high in the Ainu (25.5 %), Negidal (21.2 %) (Starikovskaya et al., 2005), Koryak (5.7 %), Even (8.1 %), and Eastern Evenk (8.9 %) populations (Derenko, Malyarchuk, 2010). However, the frequency of this mtDNA line in other Asian populations is significantly lower and decreases as one moves away from the territories where the main carriers of this line reside. The origin of these specific mtDNA lines is associated with the lower reaches of the Amur River and Sakhalin.

The distribution of the Y1a1 mitochondrial DNA (mtDNA) subgroup is limited to the Northeast Asian region. All the mtDNA lines found in the Koryak, Even, Itel’men, Negidal, Nivkh, Orok, and Ainu populations belong exclusively to this subgroup (Horai et al., 1996; Schurr et al., 1999; Bermisheva et al., 2005; Starikovskaia et al., 2005; Derenko, Malyarchuk, 2010). The main area of this mtDNA line and the frequencies of its sublineages correlate well with the distribution of the C2a1 Y-chromosome haplogroup. This is an example of parallel expansions of Y- and mtDNA haplogroups in the same region. These findings are consistent with previous research on ancient genomes from the Amur River basin, which formed a distinct genetic cluster including both ancient and modern populations from the region (people speaking Tungusic languages and Nivkh) (Wang et al., 2021).

The purpose of this study is to conduct a comprehensive analysis of the genetic structure of the Nivkh population in comparison with other indigenous populations of Siberia and the Far East. In order to address questions about the genetic affinity of the Nivkhs to other indigenous groups, we have performed genotyping for a wide range of autosomal markers on high-density DNA microarrays, as well as for a larger set of SNPs and STR markers on the Y chromosome, in various ethnic groups such as the Udege, Chukchi, Koryak, Yakut, Evenk, Buryat, Tuvinian, Khakass, Southern Altai, Ket, Chulym, and Khant.

## Materials and methods

The research material consisted of DNA samples from men and women from the Nivkh population (N = 155) living in the settlements of Nekrasovka and Moskalvo, in the Okhinsky district of the Sakhalin region. Venous blood was collected from donors in accordance with the written informed consent procedure for conducting the study (Protocol No. 10 of the Biomedical Ethics Committee of the Research Institute of Medical Genetics, dated 02/15/2021). For each donor, a questionnaire was completed with a brief family history, indicating ethnicity, ancestral place of birth, and other relevant information. Individuals were assigned to an ethnic group based on their own ethnic identity, their parents’ ethnic background, and the place of their birth.

52 DNA samples from the Nivkh population were used to analyze Y-chromosomal haplogroups and haplotypes in men. For high-density genotyping, unrelated Nivkh samples (N = 13) without intermarriage with other ethnic groups were selected. This small number of samples is due to the significant proportion of interethnic marriages among the collected individuals over the past few generations, as well as the relatively small size of the Nivkh population and the presence of close relatives on both the maternal and paternal sides in the samples.

Other populations of the indigenous people of Siberia included in this study are represented by the Udege (N = 15), Koryaks (N = 20), and Chukchi (N = 25). Samples of the Udege were collected from the villages of Krasny Yar and Agzu, in the Pozharsky and Terneysky districts of Primorsky Krai, respectively. The Koryak samples were collected in the Koryak Autonomous Okrug in the Kamchatka region, and the Chukchi samples were collected from various settlements in the Chukotka and Chukotkan Autonomous Okrugs, including the coastal regions of Lorino, Sireniki, Yanaryk, and Novoe Chaplino. Southern Altaians were also included in the study, with samples collected from the Beshpeltir (N = 24) and Kulada (N = 25) villages in the Chemalsky and Ongudaysky districts, respectively. Finally, the Ket samples were collected (N = 15) in the Kellogg settlement of the Turukhansky district in the Krasnoyarsk region; other samples were collected in Tomsk Tatar (Chernaya Rechka, Eushta, and Takhtamyshevo in the Tomsk area, N = 20), Tuvinian (Teeli in Bai-Tayga kozhuun, N = 28), Buryat (Aginskoe in the Aginsky district N = 23 and Kurumkan in the Kurumkan district, N = 28), Khanty (Kazym village in the Beloyarsk district N = 30 and Ruskinskaya in Surgut district N = 26), Khakass (Tashtypsky, N = 29 and Shirinsky N = 26 districts), Chulym (N = 22), Evenk (Zabaikalsky villages Chara, Moklakan, and Tupik, N = 25; Y – Evenks of Yakutia, N = 28), Yakut (Ust-Aldan district village Cheriktey, N = 26) settlements. The material is stored in the bioresources collection “Biobank of the Population of Northern Eurasia”.

Genome-wide genotype data were obtained using the Infinium Multi-Ethnic Global 8 microarrays (Illumina), which include over 1.7 million single nucleotide polymorphisms (SNPs). Clustering of the SNP genotype array and quality control were performed using a protocol developed by Y. Guo et al. (2014), using GenomeStudio software (Illumina GenomeStudio, version 2.0.3). A standard set of tools, including vcftools, bcftools, and plink, were used for filtering, normalization, and calculation of standard genomic statistics and metrics.

The Refined IBD algorithm (Browning B.L., Browning S.R., 2013) was used to analyze cluster blocks that are identical in origin. This algorithm produced more accurate results than the algorithms built into plink. The genotypes had been previously phased using the Beagle 5.1 software (Browning S.R., Browning B.L., 2007). To compare populations, we obtained the sums of the average lengths of clusters that were identical in origin between pairs of individuals.

PCA was used to analyze genetic relationships between populations. The NGSadmix technique (Skotte et al., 2013) and ADMIXTURE program (Alexander et al., 2009; Alexander, Lange, 2011) were used to determine the component composition and amount of impurities in individuals and populations.

To study the composition and structure of the Y chromosome, two systems of genetic markers were used in the study: diallelic loci represented by single nucleotide polymorphisms (SNPs) and polyallelic microsatellites with high variability (YSTRs). Using 589 SNPs, men were classified into different haplogroups. Genotyping of SNPs was performed using the polymerase chain reaction (PCR) method and subsequent analysis of DNA fragments through RFLP analysis (restriction fragment length polymorphism). For specific terminal SNPs, a small number of samples were genotyped for individual sub-haplogroups according to their YSTR haplotypes, and the results were obtained through NGS (next-generation sequencing) of the Y chromosome. Haplogroups were designated based on the ISOGG (International Society of Genetic Genealogy) 2019 Y-DNA Haplogroup Tree classification. Analysis of STR haplotypes within haplogroups was carried out using 44 STR markers of the non-recombining part of the Y chromosome (DYS19, 385a, 385b, 388, 389I, 389II, 390, 391, 392, 393, 426, 434, 435, 436, 437, 438 , 439, 442, 444, 445, 448, 449, 456, 458, 460, 461, 481, 504, 505, 518, 525, 531, 533, 537, 552, 570, 576, 635, 643, YCAIIa, YCAIIb, GATA H4.1, Y-GATA-A10, GGAAT1B07). STR markers were genotyped using capillary electrophoresis on ABI Prism 3730 and Nanofor-05 devices.

Experimental studies were conducted at the Center for Collective Use of Scientific Research Equipment “Medical Genomics”, which is part of the Research Institute of Medical Genetics at the Tomsk Scientific Center. The median networks of Y-chromosome haplotypes were created using the Network v10.2.0.0 software (Fluxus Technology Ltd.; www. fluxus-engineering.com), using the Bandelt median network method (Bandelt et al., 1999). The age of the haplotype diversity observed in haplogroups was estimated using the ASD method (Zhivotovsky et al., 2004), based on the average square difference in the number of repeats between all markers.

## Results and discussion

After processing the data based on the results of the microarray study to filter the genotyped samples and carry out further calculations, a search was carried out among the Nivkhs for mestizos using the NGSadmix program. The NGSadmix method, when launched on the data array we generated, showed that all samples of pure Nivkhs do not have crossbreeding with other peoples, which coincides with the results of their survey.

**Genetic relationships of the Nivkhs with the peoples of Eastern and Northeastern Siberia**

**Fig. 1.**
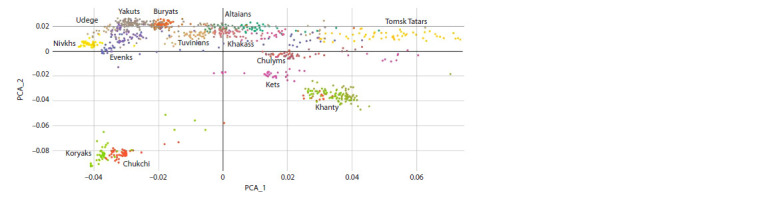
Differentiation of genomes of the population of the Far East and Siberia according to two PCA components.

When analyzing data on the frequencies of autosomal SNPs using the PCA method at the level of individual samples (Fig. 1), it is clear that the Nivkhs are closest to the Udege, as well as to the Evenks from Transbaikalia and Yakutia. The Chukchi and Koryaks are very distant from all other populations in the figure, which is consistent with their strong geographic isolation in Northeastern Siberia. It is PC2 that separates them from all analyzed samples, but according to PC1 they are very close to the Nivkhs and Udege. Their strong distance from more southern peoples indicates the presence in the Chukchi-Koryak gene pool of an older, specific genetic component associated with the aboriginal Paleolithic population of the territories where they lived. The Far Eastern samples are divided in full accordance with the territories of their residence into the northern group of the Chukchi and Koryaks and the southern group including the Nivkhs and Udege. The Evenks from Transbaikalia and Yakutia are also close to each other. The Yakuts and Buryats are a little more remote. The distance between the Nivkhs and all other populations in the figure coincides with their geographic location. The Nivkhs, Udege, Chukchi and Koryaks make up the Far Eastern group of populations, with the Nivkhs and Udege showing the greatest kinship.

Almost all samples of individual ethnic groups form specific clusters (Fig. 1), which can partially overlap in this figure, with the exception of the Tomsk Tatars, who have a fairly heterogeneous composition of the gene pool (Valikhova et al., 2022). In the three-component analysis and in the t-SNE plot, all ethnospecific clusters are much more distant from each other. Individual samples from different samples that stand out from these general groups show crossbreeding when analyzed by the NGS-Admix method, which affects their location on the graph.

**Component composition of the gene pool of populations**

**Fig. 2.**
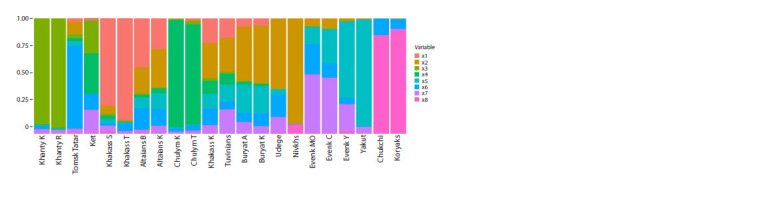
Ordered picture of Admixture components when ranking Siberian populations from west to east, K = 8.

To determine the genetic components in the gene pool of the studied populations, the Admixture program was used, which makes it possible to identify the heterogeneity of the component composition of the genome of individuals based on genotype data and accurately determine their distribution at the level of populations and individual samples. When setting the number of ancestral components to more than four, in most studied populations a genetic component specific to the Nivkhs is revealed, most clearly manifested in the analyzed array of population samples at K = 8, which can be interpreted as the “Sakhalin-Amur” genetic layer in the gene pool of modern populations (Fig. 2).

At K = 8, this component completely dominates among the Nivkhs (0.92) and Udege (0.61), and is found among the Buryats (0.50), Altai-Kizhi (0.34), Khakass-Kachins, Tuvinians (0.30), Altaians of the village of Beshpeltir (0.23), Tomsk Tatars (0.11), Evenks (0.02–0.09), Khakass Sagais (0.07) and Yakuts (0.01). It is possible that this genetic layer is associated with an ancient substrate in these populations.

**Fig. 3.**
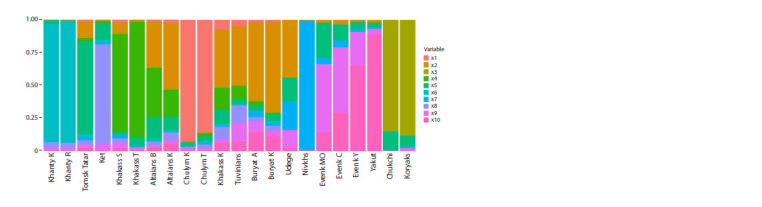
Ordered picture of Admixture components when ranking Siberian populations from west to east, K = 10.

At K = 10, a more detailed separation occurs (Fig. 3): the Nivkhs have a component specific to them (0.98), highlighted in blue in Figure 3, which is present among the Udege (0.22), and to a small extent among the Transbaikal Evenks (0.05), the Evenks of Yakutia, Khakassians, Tomsk Tatars and Buryats (0.02). The dominance in the frequency of this component in all Nivkh samples confirms that their ancestors had no contact with other peoples for quite a long time and lived in isolation on Sakhalin Island. The data obtained prove that the indigenous population of Sakhalin did not mix with other ethnic groups for a long time.

**Blocks identical by descent**

Coincidence analysis was carried out at the individual and population levels to assess common ancestry DNA blocks. A fragment that has identical nucleotide sequences in different people is the legacy of their common ancestor. The size of this IBD segment is comparable to the number of generations due to chromosome recombination during the formation of germ cells. The use of information about these genomic regions of common origin at the level of individuals and populations makes it possible to quantify the degree of genetic relatedness between people and provides additional information about the genetic connections of populations (Gusev et al., 2012).

**Fig. 4.**
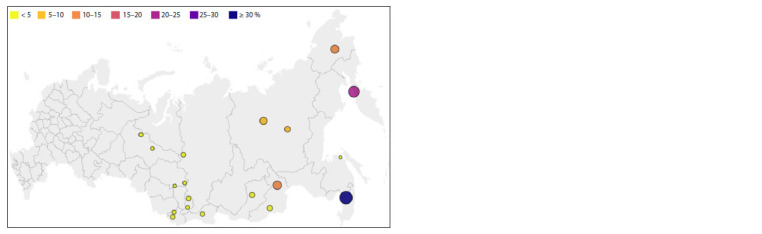
Sum of segment lengths IBD >1.5 cM between pairs of Nivkh individuals and Siberian populations

The genotypes of the Nivkhs showed a coincidence in IBD blocks with each other >1.5 cM (11 %), then with the Udege (0.58 %), Koryaks (0.47 %), Evenks (0.28 %) and Chukchi (0.18 %). With other Siberian populations, their share is much lower (Fig. 4). The agreement between the Nivkhs and other studied populations is the lowest compared to other ethnic groups. This confirms their very long isolation and lack of contact with other peoples. The proportion of interpopulation IBD blocks between the Nivkhs, Udege, Koryaks, Chukchi, and Evenks is consistent with the results of PCA and Admixture. Analysis of IBD within the Nivkh, Koryak and Chukchi populations showed that they have more common IBD than people from other samples. At the same time, among the Chukchi (55 %), Koryaks (57 %) and Nivkhs (59 %), the greatest contribution is made by short IBD fragments, which may indicate a “bottleneck” in the past during migrations to the north and northeast or isolation from other populations inhabiting the territory of Siberia.

**Genomic inbreeding coefficient**

When estimating the genomic inbreeding coefficient for ROH lengths >1.5 Mb, the Nivkhs have a relatively low level of consanguinity (FROH = 0.0268). Among the Koryaks (FROH = 0.0446) and Chukchi (FROH = 0.0431), it is maximum for Siberian populations and is almost twice as high as their average value in the territory of Siberia and the Far East. For the Nivkhs, Chukchi and Koryaks, a significant increase in the total length of the average ROH class per individual has been shown compared to other populations. This adds to the comparison with the short ROH class in Siberian populations. The results obtained indicate a relatively small number of ancestral groups of these peoples over many generations and marriage contacts between relatives, as well as a possible “bottleneck” effect. The level of homozygosity in the genomes of representatives of these Far Eastern peoples shows the highest level of inbreeding among all indigenous Siberian peoples. They have long homozygous stretches for all ROH length categories in most samples examined. These results confirm the relatively small size of their ancestral groups over a long period of time and their territorial isolation, which precluded mixing with other populations

**Y-chromosome**

The results of genotyping of SNP and YSTR markers and determination of Y-chromosome haplogroups in all samples of Nivkh men have been shown to match the data of their questionnaires on the paternal side. All men who are mestizos with Eastern European peoples on their father’s side belong to specific European sublines of haplogroups E, I1, N1a1, N1a2 and R1a1. Haplogroups of mestizos with Koreans and Orochons belong to the East Asian variants of the C2, O1 and O2 clades. All other Nivkh samples belong to sublines of three haplogroups specific to them.

The most common haplogroup among the Nivkhs is C2a1 (86 %). With such a high frequency, this Y-chromosomal line has not been recorded in any of the analyzed ethnic groups, and is maximum in purebred male-line Nivkhs compared to other peoples. It is a substrate element of their gene pool, associated with autochthonous population groups of the Okhotsk region.

Of the 37 Nivkh men without paternal crossbreeding, 16 people belong to the C2a1a subline (B90, Z32902, Z32912, Z32919, Z32926, Z32937 (xB93, Z32958)) (see the Table). The age of this lineage was previously determined to be 4,216 years (3,700–4,667) (Liu et al., 2021). This branch forms a special cluster of YSTR haplotypes, characterized by a reduction to ten in the number of tandem repeats in the DYS389I locus, specific for the Nivkhs and Koryaks. The parallel line C2a1a-B93 is also present among the Evenks, Evens, Koryaks, Yukaghirs and Yakuts. Among the Yakut Evenks and Yukaghirs, it is 15–20 %. In a very large sample of Yakuts, only four samples belong to it. One example of this branch has also been found among the Transbaikal Evenks. The presence of a specific branch C2a1a2b (B93) in these populations is associated with the ancient indigenous populations of the Amur and Okhotsk regions, which separated from the Asian ancestors from more southern regions a long time ago. According to the research team from Tartu (Karmin et al., 2015), three samples of Koryak men belonging to haplogroup C3c2 have haplotypes almost completely identical to our samples from this line. In two Evenks from Mongolia (Liu et al., 2021) and one from Russia, the C2a1a2b–B90 subline was also discovered (Karmin et al., 2015). This branch is related to C2a1a2b–M86, which previously split with the C2a–M48 branch about 11.6 Kya (Liu et al., 2021). Its spread in Eastern Siberia is associated with the relatively recent migration of Tungus tribes from the Amur region and Manchuria. The Nivkh-specific subline C2a1a2b (xB93) separated from the common ancestor even before the formation of the B93 mutation among the Tungusic peoples.

**Table 1.**
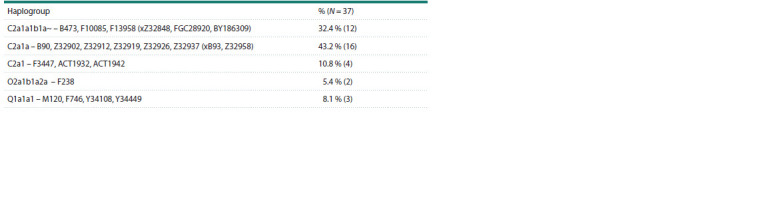
Frequencies of Y-chromosome haplogroups among the Nivkhs

The second most common line among the Nivkhs is C2a1a1b1a~ F13958 (32.4 %). This line was found in one Kazakh and three Kyrgyz, but in terms of haplotypes they differ significantly from the Nivkhs. According to the YFull website, the age of its common ancestor is 4,300 years (CI: 5,200–3,500). The C2a1 lineage (F3447, ACT1932, ACT1942) includes four Nivkhs. According to the YFull website, the age of its common ancestor is 16,000 years (CI: 17,300–14,800). This line of ancient origin was found in two Chinese people from Liaoning Province, a Korean and a Japanese person.

The large diversity of C2a1 lines among the Nivkhs and their age indicate a very early appearance of this haplogroup in the indicated territory. The spread of this line during the formation of the gene pool of the ancient population of Northeast Asia is associated with the early migrations of Mongoloid tribes. Thus, C2a1 is a marker for the settlement of the ancestors of modern northern continental Mongoloids and their further differentiation in Siberia, as well as the second wave of settlement of America, the representatives of which retained the morphological features of the ancient proto-Mongoloids of Asia

In general, the Nivkh gene pool, in terms of autosomal SNPs and Y-chromosome haplogroups, on the one hand, occupies an intermediate position between the gene pools of the Koryaks and Udege; on the other hand, it is less diverse in composition and is distinguished by the presence of three specific variants. The highest frequency of haplogroups C2a1a–B90 (xB93) among Siberian populations makes it a unique object for studying the Paleolithic layers of the total Far Eastern gene pool and reconstructing the earliest stages of human settlement of Northeast Asia.

**Fig. 5.**
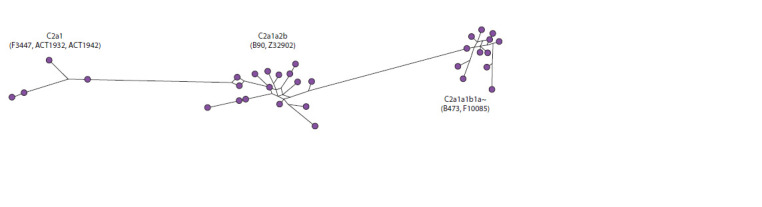
Median network of YSTR haplotypes of haplogroup C2a1 in the Nivkhs.

The overall median network of haplotypes of haplogroup C2a1 is very branched, and consists of three clusters of haplotypes that match the genotypes of terminal SNPs for these sublineages (Fig. 5). This corresponds to an estimate of the time of their separation. All three clusters demonstrate the presence of common male ancestors, the descendants of which are all analyzed Nivkh samples.

Thus, the populations that brought haplogroup C2a1 to the territory of the Amur region and Kamchatka apparently migrated north along the Pacific coast. The greatest haplotype diversity of C2a1 in the Far East indicates a significantly earlier appearance of this haplogroup in this territory, in comparison with Southern Siberia. The spread of this line during the formation of the gene pool of the ancient population of North Asia is apparently associated with the migrations of Mongoloid tribes that formed the Central Asian, Baikal and Arctic groups of anthropological types.

Two Nivkhs have haplogroup O2a1b1a2a – F238 (see the Table). It is represented among residents of China and one person from Myanmar. The age of its common ancestor is 7,500 years (CI: 8,600–6,400). Three more Nivkhs belong to the rare line Q1a1a1 – M120, F746, Y34108, Y34449, to which one Koryak, an Evenk from Yakutia and four Yukaghirs belong.

## Conclusion

The spread of C2a1 carriers undoubtedly occurred with the assimilation of the more ancient local population. Thus, the Nivkh gene pool is quite specific in the composition of Y- chromosome and mtDNA haplogroups, but very similar in autosomal markers. The results of the analysis of the samples indicate a close genetic relationship of the Nivkhs with the Koryaks, Chukchi, Udege and Evenks. The specificity of the Y-chromosome sublines and YSTR haplotypes proves that the Nivkhs had no contact with other ethnic groups for a long time and lived in relative isolation for many centuries. The results of microarray analysis also confirm this. Data on the Nivkh gene pool complement the results of paleogenetic, linguistic, anthropological and ethnological research areas. According to ethnogenesis, the Nivkhs are Paleo-Asians. It was on their genetic substrate that other Amur peoples were later formed, which is in good agreement with the results of this study of their gene pools.

## Conflict of interest

The authors declare no conflict of interest.
